# Supplementary material for: Decoding the physics of observed actions in the human brain
Source: eLife. 2025 Feb 10;13:RP98521. doi: 10.7554/eLife.98521 (PMC11810105; doi:10.7554/eLife.98521)
Supplement: Supplementary file 1. — Verbal descriptions of each participant and mean confidence ratings (from 1 = not at all to 10 = very much ± standard deviations). [file elife-98521-supp1.docx]

| Subject | break | hit | ingest | move | squash |
| --- | --- | --- | --- | --- | --- |
| 1 | splitting | hitting | swallowing | moving | hitting |
| 2 | dividing | explosion | shrinking | passing | colliding |
| 3 | break | kinetic energy | suction | touch | crash |
| 4 | break | press | eating | press | push |
| 5 | divide | crash | swallow | push | turn |
| 6 | divide | crash | sucking | pushing | squeeze |
| 7 | break | accident | consume | push | hit |
| 8 | splitting in two | pushing and splitting | shrinking | slowly pushing | pushing |
| 9 | destroying | banging | introjection | hitting | squishing |
| 10 | division | division | absorbing | pushing | impacting |
| 11 | dividing | force | absorption | action reaction | squeezing |
| 12 | divide | collide | absorb | activate | squeeze |
| 13 | crash | collision | eating | push | push |
| 14 | separation | cooperation | absorption | cause and effect | force |
| confidence | 7.0 ± 2.1 | 6.1 ± 2.9 | 7.8 ± 2.1 | 6.0 ±2.1 | 7.7 ± 2.5 |
