# Supplementary material for: Decoding the physics of observed actions in the human brain
Source: eLife. 2025 Feb 10;13:RP98521. doi: 10.7554/eLife.98521 (PMC11810105; doi:10.7554/eLife.98521)
Supplement: Supplementary file 2. — Verbal descriptions of each participant and mean confidence ratings (from 1 = not at all to 10 = very much ± standard deviations). [file elife-98521-supp2.docx]

| Subject | break | hit | ingest | move | squash |
| --- | --- | --- | --- | --- | --- |
| 1 | fix | move | move | put | act |
| 2 | shake | read | drink | take | squeeze |
| 3 | celebrate | knife | dancing | designing | pooping |
| 4 | dropping | pushing aside | lifting | putting | shitting |
| 5 | crushing | swapping | raising | putting | crushing |
| 6 | crank | swipe | raise | place | pressing |
| 7 | no idea | turning page | drinking | taking | knitting |
| confidence | 4.1 ± 3.6 | 3.0 ± 2.8 | 5.1 ± 3.1 | 4.0 ± 3.6 | 3.5 ± 3.1 |
