# Supplementary material for: Decoding the physics of observed actions in the human brain
Source: eLife. 2025 Feb 10;13:RP98521. doi: 10.7554/eLife.98521 (PMC11810105; doi:10.7554/eLife.98521)
Supplement: Supplementary file 3. — Verbal descriptions of each participant and mean confidence ratings (from 1 = not at all to 10 = very much ± standard deviations). [file elife-98521-supp3.docx]

| Subject | break | hit | ingest | move | squash |
| --- | --- | --- | --- | --- | --- |
| 1 | breaking | moving away | drinking | putting | compressing |
| 2 | breaking | wiping | drinking | relocating | squeezing |
| 3 | twist | pushing | drinking | carrying | compressing |
| 4 | break | push | drink | replacing | squeeze/press |
| 5 | break | push | drink | move | squeeze |
| 6 | breaking | pushing | drinking | pretending | pretending |
| 7 | break | shove | drink | put | smash |
| 8 | bending | sweeping sth away | drinking cup | replacing object | squishing object |
| 9 | smashing | putting aside | drinking | counting | squishing |
| 10 | lowering | push | drinking | put | folding |
| 11 | bending | pushing | drinking | grabbing | squeezing |
| 12 | break | let go | drink | put | compress |
| 13 | breaking | pushing | drinking | relocation | squeezing |
| 14 | squeezing | dusting | drinking | putting down | smushing |
| confidence | 8.0 ± 2.2 | 6.9 ± 1.9 | 9.3 ± 1.4 | 8.1 ± 2.4 | 8.3 ± 1.9 |
